# Non-Obese Type 2 Diabetes with a History of Being an Extremely Preterm Small-for-Gestational-Age Infant without Early Adiposity Rebound

**DOI:** 10.3390/ijerph19148560

**Published:** 2022-07-13

**Authors:** Nobuhiko Nagano, Chizuka Kaneko, Shoko Ohashi, Megumi Seya, Itsuro Takigawa, Ken Masunaga, Ichiro Morioka

**Affiliations:** 1 Department of Pediatrics and Child Health, Nihon University School of Medicine, Tokyo 173-8610, Japan; nagano.nobuhiko@nihon-u.ac.jp (N.N.); shoko_ohashi@tmhp.jp (S.O.); seyamegu@gmail.com (M.S.); simanerima@gmail.com (I.T.); ken_masunaga@tmhp.jp (K.M.); 2 Division of Diabetology, Endocrinology and Metabolism, Showa General Hospital, Tokyo 187-8510, Japan; cc_smileandjoy_42x80@yahoo.co.jp; 3 Department of Neonatology, Tokyo Metropolitan Ohtsuka Hospital, Tokyo 170-8476, Japan

**Keywords:** body composition, body mass index, insulin resistance, muscle mass, obesity

## Abstract

Adiposity rebound (AR), which is defined as a situation in which the body mass index (BMI) starts to increase after infancy, is a predictive marker of future development of type 2 diabetes. The patient was a 20-year-old male. He was born at 28 gestational weeks with a birthweight of 642 g (−3.20 standard deviation, small-for-gestational age [SGA]). AR during early childhood or obesity in later childhood was not observed. At the onset of type 2 diabetes (20 years of age), his BMI, body fat percentage, and body fat mass were within normal ranges (20.4, 18.4% and 10.8 kg, respectively). However, his muscle mass was 44.7 kg, with low muscle mass of the trunk and upper limbs, which was lower than the standard reference, indicating that myogenic insulin resistance was involved in the development of non-obese type 2 diabetes. This case report describes a patient with no presentation of AR and obesity during childhood, who was born extremely preterm SGA, developed non-obese type 2 diabetes with low muscle mass. We suggest that patients born extremely preterm SGA should be carefully observed for the development of type 2 diabetes, even if they did not have AR in early childhood or had not become obese.

## 1. Introduction

Small-for-gestational-age (SGA) infants, who have restricted growth in utero resulting in low birthweight, are at high risk of lifestyle-related diseases, such as hypertension, hyperlipidemia, and type 2 diabetes mellitus in adulthood after developing obesity [1]. Predictive markers in childhood have attracted attention and have been studied worldwide to prevent lifestyle-related diseases. Adiposity rebound (AR), which is defined as a situation in which the body mass index (BMI) starts to increase after infancy, has been found to be a promising factor for prediction [2,3,4,5,6,7,8,9]. The early timing of AR has been reported to be related to the development of obesity, metabolic syndrome, and type 2 diabetes mellitus [2,3,4,5,6,7,8], especially when AR occurs before the age of five years (early AR) [3]. For example, Barker et al. reported that low BMI until two years of age and marked increase in BMI after two years of age constituted high-risk conditions for ischemic heart disease in adults [2]. Arisaka et al. reported that children with AR before three years of age have increased insulin resistance at 12 years of age, compared with those with AR after three years of age [8].

Our previous study found that growth patterns during infancy and early childhood in SGA infants differed by gestational age at birth [9]. We suggested that early AR may be a predictive marker for the development of obesity, metabolic syndrome, or type 2 diabetes in term SGA infants, but not preterm SGA infants. We hypothesized that patients born preterm SGA develop lifestyle-related diseases without presenting early AR.

We report here the case of a 20-year-old male patient who developed non-obese type 2 diabetes and was born as an extremely preterm SGA infant. This patient did not exhibit early AR and developed non-obese type 2 diabetes due to low muscle mass. This observation was different from previous reports where patients with early AR developed obese type 2 diabetes due to increased fat mass [2,3,4,5,6,7,8].

## 2. Case Presentation

### 2.1. Clinical Course and Condition

The patient was a Japanese 20-year-old male. His blood type was type A and Rhesus D positive. He was born by Cesarean section at 28 weeks and two days of gestation as an SGA infant. His birthweight was 642 g (−3.20 standard deviation [SD]), and his birth height and head circumference were 31.5 cm (−2.38 SD) and 24.2 cm (−0.87 SD), showing an asymmetrical SGA. SGA was caused by hypertensive disorders during pregnancy. Severe hypoglycemia or hyperglycemia was not observed during his stay in the neonatal intensive care unit. Regarding diseases related to extremely preterm birth, retinopathy of prematurity recovered without any treatment. No chronic lung disease developed. The head magnetic resonance imaging and auditory brainstem response test were normal at term-equivalent age. At three years of age, the patient’s developmental quotient, according to the Kyoto scale of psychological development, was 91 (standard range: ≥85). At six years of age, his intelligence quotient, measured using the Wechsler intelligence scale for children-III, was 82 (standard range: ≥80).

**Figure 1:**
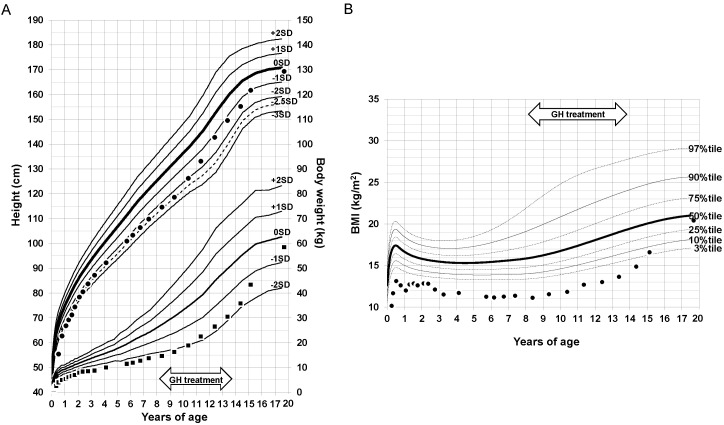
Growth curve of height and body weight from birth to the onset of diabetes. (**A**) Height and body weight. ●: height of the patient, ■: body weight of the patient. (**B**) Body mass index. ●: BMI of the patient. BMI, body mass index; GH, growth hormone; SD, standard deviation.

The patient’s height and body weight growth curve and BMI transition are shown in Figure 1A,B. Early AR or obesity was not observed in his infancy, childhood, or school age. Because of the severe short stature in children born SGA, the patient received growth hormone (GH) treatment from 8 to 14 years of age. Effective height responses were observed, and no abnormal blood glucose levels were found during the GH treatment. After discontinuing the treatment, the patient’s body weight and BMI gradually increased and type 2 diabetes without obesity developed.

The patient visited his family physician with a primary complaint of pain when urinating. A urinalysis revealed positive urine glucose levels (4+); hence, he was referred to a diabetologist. At the time of admission (20 years of age), the patient’s height, body weight, and BMI were 169.3 cm, 58.5 kg, and 20.4, respectively. His blood pressure was 101/70 mmHg; the detailed laboratory examination results are shown in Table 1. His fasting blood glucose and HbA1c levels were 175 mg/dL and 11.6%, respectively. The serum total cholesterol and non-high-density lipoprotein cholesterol levels were high (223 mg/dL and 172 mg/dL, respectively). Serum liver enzyme levels and renal function levels were within normal ranges. The insulin response on a glucagon load test and urinary storage connecting peptide immunoreactivity test was normal. The results were negative for anti-glutamic acid decarboxylase antibody and anti-insulinoma-associated antigen-2 antibody. Based on these results, the patient was diagnosed with type 2 diabetes.

The patient’s body composition analyses using an InBody s10^®^ (medical device approval number: 223AFBZX00130000; InBody Japan Inc., Tokyo, Japan) at the onset of diabetes are shown in Table 2. His body fat percentage and body fat mass were within normal ranges (18.4% and 10.8 kg, respectively). However, the patient’s total muscle mass was 44.7 kg, which was low compared with the standard range, especially those of the upper limbs and trunk. Low muscle mass without significant fat accumulation was confirmed.

### 2.2. Treatments

Soon after diagnosis, the patient started diet therapy (1760 kcal/day) and insulin therapy (38 units/day). Five weeks after starting the treatments, his HbA1c levels improved to 9.1%; insulin therapy was reduced to 26 units/day, and oral metformin administration was started (500 mg/day). Seven weeks later, the patient’s HbA1c level was 7.3% and his glycemic control further improved. His insulin dose was reduced to 17 units/day and metformin dose was increased to 1000 mg/day. Insulin therapy will be withdrawn in the future. To treat hyperlipidemia, rosuvastatin (2.5 mg/day) was also started, after which the patient’s non-high-density lipoprotein cholesterol level improved to 122 mg/dL.

## 3. Discussion

We reported a patient who was born extremely preterm SGA and developed non-obese type 2 diabetes at the age of 20 years. Surprisingly, early AR or obesity was not observed in his infancy, childhood, or school age. However, low total muscle mass, without significant fat accumulation, was noted at diabetes onset. This was a different clinical course and condition from reports of previous studies that SGA infants are more likely to develop type 2 diabetes when they become obese [2,3,4,5,6,7,8].

A relationship between the transition of BMI in childhood and the future onset of type 2 diabetes has been reported [3]. Generally, AR is defined when BMI decreases in early childhood and begins to increase at approximately five to six years of age. The appearance of AR earlier than five years of age is associated with future onset of obesity and type 2 diabetes [3]. A recent study reported that extremely preterm SGA children did not catch up in height or body weight until around six years of age [10]. In our case, the BMI remained low until the onset of type 2 diabetes, but it began developing at a young adult age. Even when early AR and obesity are not observed in extremely preterm SGA children, type 2 diabetes may develop.

Skeletal muscle is the main organ that insulin acts on to take up glucose, and accounts for approximately 70% of the total glucose processing capacity of the body [11]. Insulin resistance in the skeletal muscle results in conversion of ingested carbohydrates into lipids (de novo lipogenesis) in the liver rather than glycogen in muscle [11]. Recent reports showed that extremely preterm infants exhibited insulin resistance until adulthood [12,13]. A human study including 100 adults with an average age of 32 years who were born with an extremely low birthweight reported a four-fold risk of developing impaired glucose tolerance, compared with adults born at term [12]. Another human study involving 163 preterm-born adults found that insulin resistance was significantly higher than that in term-born adults [13]. It has been speculated that patients born preterm and with extremely low birthweight have lesser lean body mass (mainly muscle mass) than term-born patients [14]. A basic study using mice showed that dysgenesis at birth led to decreased muscle volume and degeneration of muscle composition during adulthood. This was associated with insulin resistance [15]. Our patient exhibited a normal range of fat mass but a decrease in muscle mass. Kaga et al., found that healthy non-obese males have low muscle insulin sensitivity [16]. Myogenic insulin resistance may be involved in the development of non-obese type 2 diabetes in patients born as extremely preterm SGA infants.

Tajiri et al., reported that reduction in skeletal muscle mass, especially in the lower limbs, is associated with insulin resistance in Japanese type 2 diabetes patients [17]. In this case, the muscle masses of the trunk and upper limbs were small, but the muscle mass of the lower limbs was relatively large. Chao et al., have also shown that upper limb circumference can be an indicator of insulin resistance in non-obese elderly individuals [18]. Low muscle mass of the trunk or upper limbs may also be associated with insulin resistance.

In general, the accumulation of visceral fat is more strongly associated with insulin resistance than that of subcutaneous fat [19,20]. Even if the amount of fat is the same, insulin resistance depends on the type of fat (visceral fat or subcutaneous fat). In our case, the body composition analyzer showed normal fat mass; however, the distribution of visceral fat and subcutaneous fat could not be evaluated, which is a limitation of this case study.

This case was treated with GH from 8 to 14 years of age due to severe SGA short stature. There are currently debates regarding the association between long-term GH treatment and the development of diabetes. Many studies have found that GH treatment was not related to insulin resistance or abnormal sensitivity [21,22,23]. However, some have reported that GH treatment reduced insulin sensitivity [24]. In healthy older subjects, GH administration for 26 weeks impaired insulin sensitivity in the liver, but not in skeletal muscle [25]. Although we considered that the development of type 2 diabetes was not caused by GH treatment in this case, because no abnormal blood glucose levels were found during the GH treatment and six years had already passed after the end of the treatment, long-term GH treatment might have been involved in the pathogenesis. Further studies using more patients are needed to determine this.

## 4. Conclusions

This is the first report of a patient who was born extremely preterm SGA who did not have AR during early childhood but developed non-obese type 2 diabetes with significant decrease in muscle mass. Insulin resistance due to decrease in muscle mass, not fat accumulation, might account for the pathogenesis. Patients born extremely preterm SGA should be carefully observed for the development of type 2 diabetes, even without obesity or early AR. In particular, children with SGA short stature who received GH treatment should be followed-up regularly to monitor the potential development of type 2 diabetes after discontinuation of the treatment.

## Figures and Tables

**Table 1:** Laboratory data.

Blood		Patient’s Results	Normal Range	
Complete blood count	WBC	4600	4000–10,000	/µL
	RBC	602	420–550	×10^4^/µL
	Hb	17.1	13.2–17.2	g/dL
	Ht	51.3	39.4–49.8	%
	PLT	23.0	15.0–40.0	×10^4^/µL
Liver function	AST	25	7–38	IU/L
	ALT	32	8–40	IU/L
	LDH	198	124–222	IU/L
	γ-GTP	19	0–66	IU/L
	TP	7.2	6.3–8.2	g/dL
	ALB	4.4	4.1–5.1	g/dL
Renal function	BUN	16.1	8.0–21.0	mg/dL
	CRE	0.67	0.61–1.04	mg/dL
	UA	4.4	3.7–7.0	mg/dL
	Na	141	133–147	mmol/L
	K	4.0	3.5–4.7	mmol/L
	Cl	101	98–110	mmol/L
Lipid-related test	TC	223	125–220	mg/dL
	HDL-C	51	40–86	mg/dL
	Non-HDL-C	172	90–149	mg/dL
	Triglyceride	51	30–150	mg/dL
Glucose-related test	Fasting PG	175	70–109	mg/dL
	HbA1c	11.6	4.6–6.2	%
	Glycated albumin	29.2	12.4–16.3	%
	Anti-GAD antibody	<5.0	<5.0	U/mL
	Anti-IA-2 antibody	<0.6	<0.6	U/mL
**Urine**	**Patient’s Results**	**Normal range**		
Protein	2+	-		
Glucose	1+	-		
Ketone body	±	-		
Occult blood	±	-		
Urinary storage CPR test	76.3	≥20	µg/day	
**Glucagon loading test**	**0 min**	**Normal range**	**5 min**	**Normal range**
CPR (ng/mL)	0.9	≥0.5	2.4	≥1.0

ALB, albumin; ALT, alanine aminotransferase; AST, aspartate aminotransferase; BUN, blood urea nitrogen; Cl, chlorine; CPR, connecting peptide immunoreactivity; CRE, creatinine; GAD, glutamic acid decarboxylase; GTP, glutamyl transpeptidase; Hb, hemoglobin; HbA1c, glycosylated hemoglobin; HDL-C, high-density lipoprotein cholesterol; Ht, Hematocrit; IA-2, insulinoma-associated antigen-2; IRI, immunoreactive insulin; K, potassium; LDH, lactate dehydrogenase; Na, sodium; PG, plasma glucose; PLT, platelet; RBC, red blood cell; TC, total cholesterol; TP, total protein; UA, uric acid; WBC, white blood cell.

**Table 2:** Body composition analysis.

Measurement Parameters	Results		Standard Range *
Height	169.3	cm	
Body weight	58.5	kg	
Body mass index	20.4	kg/m^2^	18.5–25.0
Body fat percentage	18.4	%	10.0–20.0
Body fat mass	10.8	kg	7.6–15.1
Total muscle mass	44.7	kg	45.5–55.7
Right arm	1.78	kg	2.55–3.45
Left arm	1.83	kg	2.55–3.45
Trunk	17.5	kg	21.5–26.3
Right leg	9.21	kg	7.51–9.17
Left leg	8.53	kg	7.51–9.17

* Standards indicate the ideal value that should be based on the standard weight to maintain the balance of body components regardless of race, age, and physique. The standard weight is calculated by height (m) × height (m) × standard BMI (male = 22, female = 21) (https://www.inbody.co.jp, accessed on 7 June 2022, In Japanese).

## Data Availability

The data that support the findings of this case study are available from the corresponding author upon reasonable request.
